# Socioeconomic Influences on Reports of Canine Welfare Concerns to the Royal Society for the Prevention of Cruelty to Animals (RSPCA) in Queensland, Australia

**DOI:** 10.3390/ani9100711

**Published:** 2019-09-23

**Authors:** Hao Yu Shih, Mandy B. A. Paterson, Clive J. C. Phillips

**Affiliations:** 1 Centre for Animal Welfare and Ethics, University of Queensland, White House Building (8134), Gatton Campus, Gatton, QLD 4343, Australia; mpaterson@rspcaqld.org.au (M.B.A.P.); c.phillips@uq.edu.au (C.J.C.P.); 2 Royal Society for the Prevention of Cruelty to Animals Queensland, Brisbane, QLD 4076, Australia

**Keywords:** dog, animal welfare, animal cruelty, RSPCA, shelter, socioeconomic

## Abstract

**Simple Summary:**

The role of the socioeconomic status of dog owners in canine welfare concerns is not fully understood. We conducted a retrospective study of 107,597 canine welfare complaints attended by the Royal Society for the Prevention of Cruelty to Animals (RSPCA) Queensland from 2008 to 2018. We explored the relationship between the owner’s socioeconomic status and reported (rather than confirmed) complaints about welfare of dogs. The socioeconomic status of the owner was estimated from the postcode of where the alleged welfare issue occurred, using government statistics for Socio-Economic Indexes of different regions of Australia. Reported complaints were correlated with socioeconomic scores. There was a lower median socioeconomic score in our study group compared to the entire Queensland state, indicating that alleged canine welfare concerns were more likely to be reported in areas with inhabitants of low socioeconomic status. The status was also low if the complaint was about a crossbred rather than a purebred dog. Among the purebred dogs, complaints involving working dogs, terriers, and utility breeds were associated with the lowest socioeconomic scores. The following complaints were associated with low socioeconomic status: cruelty, insufficient food and/or water, a dog not being exercised, a dog being confined/tethered, failure to provide shelter or treatment, overcrowding, a dog being in poor condition or living in poor conditions. Increased status was observed in alleged cases of a dog being left in a hot car unattended.

**Abstract:**

Human–dog relationships are an important contributor to the welfare of dogs, but little is known about the importance of socioeconomic status of the dogs’ owners. We conducted a retrospective study of canine welfare complaints, using Australian government statistics on the socioeconomic status of the inhabitants at the location of the alleged welfare issue. The socioeconomic score of inhabitants at the relevant postcode was assumed to be that of the plaintiff. Our dataset included 107,597 complaints that had been received by RSPCA Queensland between July 2008 and June 2018, each with the following information: the number of dogs involved, dog(s) age, breed(s), suburb, postcode, date received, and complaint code(s) (describing the type of complaint). The median index score for relative social advantage of the locations where the alleged welfare concern occurred was less than the median score for the population of Queensland, suggesting that welfare concerns in dogs were more commonly reported in areas with inhabitants of low socioeconomic status. It was also less if the dog being reported was not of a recognised breed, compared to dogs of recognised breeds. Dogs reported to be in the gundog breed group were in the most socioeconomically advantaged postcodes, followed by toy, hound, non-sporting, working dog, terrier, and utility breed groups. Reports of alleged cruelty, insufficient food and/or water, a dog being not exercised or being confined/tethered, failure to provide shelter or treatment, overcrowding, a dog being in poor condition or living in poor conditions were most likely to be made in relation to dogs in low socioeconomic postcodes. Reports of dogs being left in a hot vehicle unattended were more likely to be made in relation to dogs in high socioeconomic postcodes. It is concluded that both canine welfare complaints and dogs in specific breed groups appear to be related to the owner’s socioeconomic status. This study may be used to improve public awareness and to tailor educational campaigns toward different populations.

## 1. Introduction

Dogs (*Canis familiaris*) share intimate relationships with humans as they are one of the most popular pets in our society [1]. According to the 2016 statistics, 3,555,000 Australian households (38.5%) own at least one dog, accounting for 4,759,700 dogs in Australia [1]. In the household, owners may develop a strong attachment to their dogs and vice versa, which creates a mutually beneficial relationship [2,3]. For instance, dog ownership apparently reduces owners’ physiological and psychological stresses [3,4], as well as providing a safe haven for their dogs [5]. However, the human–dog relationship does not always provide mutual benefits and sometimes may even break down [6].

Animal cruelty refers to any morally or legally unacceptable behaviour which causes animals to experience physiological, psychological, and/or behavioural discomfort [7,8]. Animal welfare concerns are reported in different forms and in different species, and dogs are one of the most commonly reported victims [9]. Concerns may involve violence [10,11], injuries caused by their involvement in the sporting industry [12,13], abandonment [14,15], inappropriate surgical procedures [16], and neglecting care of the dog [9,17]. Not only do these issues compromise the welfare of dogs, some of these welfare concerns (e.g., intentional abuse and dog fighting) have been recognised as sentinels of other social issues [6,9], in particular domestic violence [18,19,20] and sexual assaults [21]. Some have also been reported to be a precursor of antisocial behaviours among young people [7,22,23].

To address these cruelty issues, animal protection legislation has been enacted worldwide [8,9,24]. In Queensland, Australia, animals are protected by *Animal Care and Protection Act 2001* (ACPA) [8]. This state-based legislation empowers the State to appoint inspectors, some of whom are employed by the Royal Society for the Prevention of Cruelty to Animals, Queensland (RSPCA Qld), who investigate potential breaches of the Act and enforce compliance with the Act [8]. There are two main offences under the ACPA: failure to fulfil ‘duty of care’ responsibilities and cruelty. There are a number of other specified offences. The Act recognises that a person who has charge of an animal owes that animal a duty of care. Failure to provide such care is the basis of the “breach of duty of care” offence. This offence covers such actions as not providing sufficient food, water, exercise, veterinary care, and suitable living conditions. It is not only the owner that has a duty of care towards an animal; anyone who is even temporarily in charge of an animal has this responsibility. The second major offence is “animal cruelty” and according to Section 18 of the ACPA cruelty describes any action that causes unjustifiable and unnecessary physical and mental discomfort to animals, inappropriate confinement or transport, unreasonable injuries and inhumane death [8]. A cruel act can be committed by anyone towards an animal, whether it is their own animal, another domestic animal or even a wild animal [8]. It is important to note, that under the ACPA, it is not necessary for a person to have the intention of being cruel for the offence to be proven in Queensland. If an action carried out by a person causes pain and suffering and the action was intentional, the person may be charged with cruelty. The intention to carry out the action must be proved but not the intention to be cruel. If a lack of action deprives an animal of its fundamental needs, then the person who has a duty of care towards the animal may be charged with a breach of their duty of care or cruelty depending on the circumstances. Intention may be considered during sentencing however [8]. Other offences under the Act include unreasonable abandonment or release, the carrying out of prohibited surgical procedures (e.g., tail docking, ear cropping, debarking, etc.); being involved in, or having items used for, a prohibited event, such as dog or cock fighting; and allowing an animal to injure or kill another animal [8].

Potential cases are reported to RSPCA Qld through various means. RSPCA Qld has a “Cruelty Complaints” telephone number manned 24 h a day, seven days a week and complaints also come in through emails. These complaints are primarily made by members of the public but a few are also made by veterinarians and veterinary nurses, council officers, and other government and non-government employees visiting a location as part of their duties. Animals surrendered to the RSPCA or that come in as strays may be investigated by RSPCA Qld inspectors if cruelty or neglect is suspected. All cases reported by sources mentioned above were the focus of this study.

Apart from aiding law enforcement, identification of risk factors associated with animal welfare concerns would be beneficial in the development of awareness and in education of the general public. Risk factors include dogs’ breed or breed group [12,15,25,26,27], dogs’ ages [15,28], behaviours [15,26], owners’ characteristics [15], and the complainant’s socioeconomic status [6,20,22,29]. Among all these risk factors, the role of household socioeconomic level in crimes and animal welfare concerns is not yet well understood [6,20,22]. It has been proposed that socioeconomic status is negatively correlated with the frequency of crimes, domestic violence, and neglecting and abusing animals [6,22,29,30,31]. However, a tautologous relationship has also been postulated, because a dog owner’s socioeconomic status may affect his or her ability to provide for all aspects of good welfare.

Therefore, this study aimed to investigate the relationships between socioeconomic status, dog breeds, and different types of dog welfare complaints. The socioeconomic status was quantified using a socioeconomic score derived from the postcode of where the alleged welfare issue occurred and government statistics for Socio-Economic Indexes of different postcode regions of Australia. We hypothesized that owners from relatively poor socioeconomic postcode regions would be more likely to be the subject of complaints about an absence of key resources for dogs, such as insufficient food, water, living space or veterinary care, lack of shelter, and poor living conditions. We also hypothesized that complaints concerning owners from poor socioeconomic regions would relate more to dog fighting, because of a known potential association with low socioeconomic status [32]. Finally, some research suggests that low socioeconomic status people are less likely to travel with their dogs, and therefore it is possible that low socioeconomic status is less likely to relate to dogs being left in a hot vehicle [33]. This is the third report in a series relating to the analysis of RSPCA Qld canine welfare complaint data [see also 27,28].

## 2. Materials and Methods

From July 2008 to June 2018, RSPCA Qld received 129,036 canine welfare complaints. Some involving more than one dog were recorded as multiple complaints sharing the same case number, while others were recorded as one complaint with multiple animals. To avoid sample bias due to multiple entries, we only retained the first complaint of case numbers with multiple entries, discarding 21,439 entries as a result. There remained 107,597 canine welfare complaints for this retrospective study. The data analysis was originally undertaken on the entire dataset and then repeated with the reduced number. Finding the complaint distribution and demographics to be similar, we opted for the reduced dataset to avoid pseudoreplication. Animal welfare complaints that fell within the geographical zone of responsibility of RSPCA Qld (determined by a Memorandum of Understanding between RSPCA Qld and Biosecurity Queensland, the Government Department tasked with the administration of ACPA) were investigated by RSPCA Qld inspectors. All other complaints were referred by RSPCA Qld to Biosecurity Queensland to be investigated by their inspectors. All complaints received by RSPCA Qld were included in this study, regardless of which authority investigated them.

All complaints were recorded in ShelterBuddy^®^ (RSPCA, Queensland, Australia), the RSPCA Qld database. The following information was requested from the reporter of each incident at the time of taking the complaint: the number of dogs involved and their age, breed(s) (if known), the “complaint code(s)”, suburb, postcode and date. All cases were investigated either by RSPCA Qld inspectors (n = 100,432) or Biosecurity Qld inspectors (n = 7165). It is recognized that some of the calls, once investigated, were found not to relate to a breach of the ACPA or to a genuine welfare concern. However, the outcome data for complaints were not analysed in this research, which focused solely on the complaint calls received by RSPCA Qld.

Dogs were classified according to two broad age ranges, dog and puppy, based on reporters’ interpretation. It was important to recognise that the information recorded from the complainant may be inaccurate or inaccurately interpreted, e.g., a small dog is commonly referred to as a puppy in Queensland. Records regarding breed and the number of dogs involved were based on either complainants’ initial reports or comments from trained inspectors, again recognising inaccuracies with identification of the breed. The “complaint code” was selected by the staff member receiving the call or email from a drop-down menu of 18 possible complaints (Appendix A) [28]. Multiple “complaint codes” were able to be selected for each case according to the description of what was alleged to have happened to the dog(s), and each was treated as a separate code for analysis.

### 2.1. Socioeconomic Scores

Australia is spatially divided into regions by postcodes; our dataset included the postcode of the location of the dog being reported, which was taken as a proxy measure for the socioeconomic status of the owner. We also reviewed the Australian government’s Socio-Economic Indexes for Areas (SEIFA) developed in 2011 [34], which rank postcode regions in Australia by the socioeconomic level of inhabitants. Four indices are assigned to each area to describe the local socioeconomic status: (1) the Index of Relative Socio-Economic Disadvantage, (2) the Index of Relative Socio-Economic Advantage and Disadvantage, (3) the Index of Economic Resources, and (4) the Index of Education and Occupation. Each index is ranked by decile, percentile, and score. Among these four indices, the Index of Relative Socio-Economic Advantage and Disadvantage (IRSAD) correlates well with the other three and is suitable for comparing the entire range of areas, and was therefore considered the most appropriate descriptor of the socioeconomic level of inhabitants of each postcode [34]. The IRSAD score is a weighted combination of selected indicators of advantage and disadvantage: household income, availability of internet connection, educational level, occupation, employment rate, property, mortgage, and health. Nationwide, the score is standardized with a mean of 1000 and a standard deviation of 100, with a mean, median and standard deviation in Queensland of 999, 1014 and 65 respectively. An area receives a score of 1000 if all of the above indicators are equal to the national average; the score for an area increases or decreases if the indicators are greater or less than the national average, respectively [34]. The index is positively associated with socio-economic advantage and negatively associated with socio-economic disadvantage, thus a region with a high IRSAD score is more likely to have people with high socioeconomic levels dwelling within it [34].

### 2.2. Dog Breeds

Any breed in our dataset that was recognized by the Australian National Kennel Council (ANKC) [35], New Zealand Kennel Club (NZKC) [36], American Kennel Club (AMKC) [37], or United Kennel Club (UKC) [38] was considered a recognized breed (RB) (see Appendix B list of recognized breeds). Any other reported breed in our data was considered an unrecognised breed (UB), including all crossbred dogs without any identified breed. If more than one dominant breed was listed, the first mentioned was used. For instance, Great Dane × Bull Arab was categorized as Great Dane (Appendix B).

To achieve a secondary representation of breed recognition, RB breeds were amalgamated into the following seven breed groups based on the breed inclusion categories of the ANKC: toys, terriers, gundogs, hounds, working dogs, utility, and non-sporting. Breeds not listed by the ANKC but recognised by the NZKC, AMKC, or UKC were categorized into one of the seven groups based on the description of each kennel club. Some breeds (e.g., Australian Koolie and Bull Arab) were listed by the council registration, it being an obligation of all dog owners in Queensland to register their dogs with the local council. As a result, they were on the breed list (Appendix B), however they were not recognised as breeds by any major kennel club worldwide. Therefore, these breeds were categorized as unrecognized breeds, UB. If the breed description was left blank, the dogs’ breed was considered unknown (n = 15,576/107,597), and these complaints were excluded from any data analysis related to breed factors. Our previous study suggested that, compared to specific breeds, breed groups and the dichotomization into RB and UB provided better agreements between the public and the trained RSPCA inspectors, and were therefore used for genotype identification in a public reporting system [27]. Therefore, this study used breed groups and RB/UB dichotomization rather than specific breeds for statistical analyses.

### 2.3. Statistical Analysis

Data was analysed using the statistical package Minitab^®^ 17.3.1. (Minitab, LLC., State College, PA, USA). The distributions of IRSAD scores of both our entire study group and the RB/UB differentiation were not normal. Box–Cox and John transformations were used, but the assumption of normal distribution of residuals still could not be met. Therefore, a one-sample sign test was used to compare the median IRSAD scores between owners of dogs involved in an alleged incident in our dataset and the entire Queensland population as recorded by SEIFA. A Mann–Whitney test was conducted to compare the IRSAD scores of postcodes where there had been reports of RB and those of UB. As for the IRSAD scores of owners of dogs of the different breed groups, normality was assessed by visual inspection of residual plots. All complaints were independent but the assumption of equal variance could not be met based on the Bartlett’s test (*p* = 0.018). Consequently, Welch’s ANOVA followed by the Games-Howell pairwise comparisons were used to compare IRSAD scores between dogs of the different breed groups.

To determine factors influencing complaint codes, the IRSAD score, dog’s age (dog or puppy), and breeds (UB or RB) were entered into eleven binary logistic regression models as fixed factors, using non-linear logit models with an alpha value for variables to enter the model of 0.15 [39]. Complaint codes were entered into the model as outcomes. Each case was independent from each other, and little multicollinearity was observed for all independent variables, with the variance inflation factors being less than 5. The linearity of the independent variable (the IRSAD score for the postcode of the dog) and the log-odds was assessed by plotting the independent variable and the log-odds fitted with a linear regression line. The assumption of linearity was considered met when the *P* value of a straight-line regression was less than 0.05. Eight complaint codes—Abandonment, Baiting/poisoning, Causing a captive animal to be injured/killed by dog, Dog fighting or other prohibited offence, Emergency relief, Keeping or using an animal for blooding/coursing a dog, Prohibition order breached, and Tail docking or other surgical procedure—did not fulfill the linearity assumption. Therefore, to fulfill the linearity assumption, Causing a captive animal to be injured/killed by dog, Dog fighting or other prohibited offence, Keeping or using an animal for blooding/coursing a dog, and Knowingly allowing an animal to kill/injure another were combined and categorized as a new code—Dog fighting. Baiting/poisoning, Cruelty, Prohibition order breached, and Tail docking or other surgical procedure were combined and categorized as a new code—Cruel act. Abandonment did not meet the linearity assumption statistically, but the graph was linear by observation, so the code was still used to construct a stepwise forward binary logistic model. Finally, the IRSAD score was removed from the regression model of Emergency relief because it did not meet the linearity assumption and the code was rarely cited in the past decade (0.01%, n = 8) [28]. Eleven stepwise forward binary logistic regression models were constructed to examine how different fixed factors (IRSAD score, dog’s age and breeds) correlated with different outcomes (9 complaint codes and 2 combined complaint codes). Separate models were constructed for each code with the same input variable. In this paper, we focused on the relationship of IRSAD scores with complaint codes and breed factors. The relationship of other variables with the complaint codes have been reported separately [27,28].

## 3. Results

### 3.1. Descriptive Statistics

The median IRSAD score of owners being reported (median = 975) was significantly lower (*p* < 0.001) than that recorded for the population of Queensland (median = 1,014), and the Q1 and Q3 values were less (Table 1). Owners of reported UB dogs had significantly lower IRSAD scores (median = 970) than those reported owning RB dogs (median = 981) (*p* <0.001) (Table 2). Mean IRSAD scores for the postcodes of the different breed groups reported were significantly different (*p* <0.001) (Table 3), with gundogs (994 ± 63.0) being the highest, followed by toy (986 ± 64.3) and hound (984 ± 63.7), then non-sporting (980 ± 64.6) and working dogs (977 ± 63.0), terriers (977 ± 62.7) and utility dogs (976 ± 62.2).

### 3.2. Complaint Codes

The IRSAD scores of postcodes of dogs who were or were not reported for each complaint code significantly differed for the nine regression models (Table 4). The odds ratio (OR) was defined as a one unit increase in the IRSAD score leading to a corresponding x-fold decrease or increase in the odds of the cited event. The following codes were associated with dogs from postcodes with low IRSAD scores: cruel act (OR = 0.9994, *p* < 0.001), insufficient food and/or water (OR = 0.9981 *p* < 0.001), no exercise/confined/tethered (OR = 0.9979, *p* < 0.001), no shelter (OR = 0.9990, *p* <0.001), no treatment (OR = 0.9986, *p* < 0.001), overcrowding (OR = 0.9982, *p* = 0.002), poor dog condition (OR = 0.9974, *p* < 0.001) and poor living conditions (OR = 0.9996, *p* = 0.002). A single code was associated with dogs in postcodes with a high IRSAD score, hot animal in a car (OR = 1.0067, *p* < 0.001).

## 4. Discussion

### 4.1. Socioeconomic Status of Dog Owners

A better understanding of relationships between the socioeconomic status of dog owners and specific welfare issues could help to elucidate the reasons for the issues, as well as helping to target specific sectors of the population for education about dog welfare. Compared to the median IRSAD score across the entire Queensland state, our study group had significantly lower IRSAD scores, indicating that the reported dogs were in postcodes with inhabitants at a lower socioeconomic level. It is possible that dog owners generally come from postcodes with inhabitants at low socioeconomic status, but because not all welfare issues were more frequently reported in lower socioeconomic regions and one was more frequent in higher socioeconomic regions, we consider that there may be an important relationship between welfare issues and socioeconomic status.

The score differences contributed by the variables appear small, and the odds ratios for IRSAD scores in the regression model were close to one. This is probably because the distribution of IRSAD scores across different regions in Queensland is narrow, with an interquartile range of 71.1 and a standard deviation of just 64.8, compared with a range of 558. Also, our dataset only covers the coastal area of Queensland which is relatively homogenous in terms of the socioeconomic level of inhabitants. The difference between our median score and that in Queensland, 38.3, represents about 59% of one SD in that region. Since about 68% of values lie within 1 SD of the mean, it can be seen that the differences in IRSAD scores in the variables tested in this study are meaningful and reflect the range in values from a significant proportion of the total Queensland population, about 40% (68% × 59%). Such a small difference is important because it should enable us to predict differences in commonly reported dog welfare concerns across populations and regions in a large and relatively homogenous area. The majority of previous studies have focused on socioeconomics and animal abuse [20,22,29]. The relationship between socioeconomic level and other welfare concerns in relation to canines [6] has received little research attention. This study may bridge this gap by determining some of the factors that relate to specific welfare concerns.

A key feature of the IRSAD score is that it might positively relate to the financial circumstances of the owner, notwithstanding the previously mentioned concern that the owner may not be represented by the status of the entire postcode, since it includes the % of people whose annual household income is < AUS $20,799 and whose rent is less than AUS $166/week [34]. This could indicate some constraints on the part of the owner in providing for the welfare of his or her dog, such as provision of adequate food, shelter, or other resources. This is discussed later in relation to individual complaint codes. The IRSAD score is also positively associated with the inhabitants’ educational level, a key component being whether the members of the household progressed past year 11 in school, assuming they were over 15 years of age. This could affect whether the owner has sufficient knowledge to care for his or her dog, providing suitable nutrition, for example. Important detractors in the IRSAD include unemployment and the percentage of employed people classified as “labourers” living in that postcode [34]. This could relate to whether the owner has sufficient financial resources to care for his or her dog. However, these results should be interpreted with caution because the IRSAD score relates to the entire postcode, which may include substantial variation within and between regions. Clearly the circumstance of the owner or owners may be different to that of other inhabitants in the postcode in question.

Broad correlations between socioeconomic status and human behaviour towards animals have been noted previously [6,40,41]. People with high socioeconomic levels are more likely to advocate for animal welfare and to volunteer for animals [40], whereas those with lower socioeconomic backgrounds are more likely to be involved in animal neglect and abuse [6]. Our findings are in agreement with these previous conclusions.

It is also important to remember that in this study we analyzed only reported data, i.e., alleged welfare issues, which may not all reflect actual cases. It is known that complainants will report a neighbour out of spite, misread a situation, or report actions which may not actually represent a breach of the ACPA. The negative correlation between the socioeconomic status of the dog’s owner and the risk of most reported welfare issues may also be explained by the fact that individuals may be more prepared or likely to report welfare issues in low socioeconomic postcodes. Since household income, home ownership, and full-time employment were reported to negatively correlate with dog ownership, it is less likely that the higher number of reports in low socioeconomic regions is because people from low socioeconomic regions own more dogs [42]. However, the same study reported living in rural locations was associated with higher odds of owning a dog [42], and we found a tendency for low socioeconomic regions to overlap non-urban regions [43]. Therefore, the socioeconomic level and dwelling in rural regions may both increase the possibility of being reported; yet we could not validate these hypotheses as the IRSAD score is a generalization of postcode regions ignoring the within postcode variation. However, inconclusive results were reported in another study, that found that ones’ personal and household income levels were not associated with the propensity of being reported for animal cruelty [44].

### 4.2. Breed

Not only can socioeconomic level of dog owners be related to animal welfare concerns, it can also be linked with breed factors. Reported cases involving UB dogs were potentially related to owners being socioeconomically disadvantaged compared with cases involving RB dogs. Similar results of breeds’ predisposition to welfare concerns and socioeconomic levels were observed when we examined the RB dogs. Reported cases involving utility breeds, terriers, and working dogs were associated with the three least socioeconomically advantaged groups. Utility breeds, terriers, and working dogs were also more commonly reported for canine welfare concerns [27]. Although this study involved only reported but not confirmed cases, these results potentially support previous studies suggesting that people with lower income have higher risks of mistreating animals [20,22], which might be further associated with specific dog breeds, and linked to some breed-specific complaints [27]. Nevertheless, it is important to note that the differences in socioeconomic scores among different breed groups were statistically significant but small, and there may be some inaccuracies and biases when breeds were reported. Therefore, over-interpretation should be avoided.

### 4.3. Complaint Types

There were clear correlations between the socioeconomic level and complaint type being reported. For example, allegedly committing cruel acts was associated with lower socioeconomic level.

Previous research focusing on crime and animal cruelty has found negative relationships between the socioeconomic level and tendencies to commit crime, including being cruel to dogs and cats [22,45]. However, a study comparing animal cruelty between rural and urban regions found that rural residents mostly targeted cats rather than dogs [10], and another study investigating community demographics of animal cruelty reports found no differences among urban, town, and rural residents in the likelihood of being reported for animal cruelty [43].

Except for canine abuse, most complaints for which the owner was from a low socioeconomic background were neglect related. The aforementioned explanation is that people with lower socioeconomic backgrounds may lack the ability (e.g., money, space, or transportation) to manage animal care and welfare [6]. Moreover, this finding is in line with a previous study that people of lower socioeconomic status tended to moralize transgressions that did not cause obvious harm to animals [46,47]. Less affluent people may therefore be more likely to view an animal welfare compromise that is not overtly cruel as a moral but not a legal issue [6,46,47]. Consequently, there is a risk that people with lower socioeconomic backgrounds may tend to neglect the fundamental needs of their dogs, including failing to provide appropriate nutrients, adequate living conditions, and medical treatments, which increases the chance of them being reported. In this respect, the first of our hypotheses was supported, that complainants from relatively poor socioeconomic postcode regions would be more likely to complain about an absence of key resources for dogs. This may relate to the factors included in the IRSAD score that are relevant to educational level. The IRSAD score of different postcodes across Australia is highly correlated with the Index of Education and Occupation score, with a Spearman’s rank correlation of 0.85 [34]. This indicates a potential relationship between the low levels of education and neglect-related canine welfare concerns, either because people who are less educated do not consider deprivation as an act of neglect, or they are simply lacking in knowledge about the welfare and care of dogs [6,29].

Among these neglect-related complaints, the finding associated with insufficient (medical) treatments seems contradictory to our previous study [27]. This study reveals that insufficient veterinary treatments were less commonly reported in regions of lower socioeconomic status, which is supported by the fact that household income limits owners’ access or willingness to provide veterinary care [48,49]. In addition, UB dogs were more commonly reported in lower socioeconomic regions. Consequently, it would be expected that UB dogs would be the subject of more complaints about poor veterinary care. However, according to our previous study, RB but not UB dogs were more likely to be reported with insufficient veterinary treatments [27]. The potential predisposition of RB dogs to a complaint about lack of veterinary care may be influenced not only by owners’ socioeconomic status, but also by other factors. These factors may include morality [10], attitudes to the welfare of breeding dogs [50], human–animal bond [49], and registration rate [51], which can outweigh or confound the effects of socioeconomic level. For instance, people who are affluent but have less moral conviction may prefer to purchase an RB dog from a breeder rather than adopting an UB dog from a shelter, and the owners with less moral conviction may be less likely to bring their sick dogs to a veterinary clinic [49]. Besides, the potentially low registration rate of UB dogs [27] may encourage owners to abandon their dogs when medical care is required, leading to a reduced but inaccurate prevalence of UB dogs being reported for lack of treatment. However, these hypotheses cannot be confirmed in this study as they were all reported not confirmed cases.

Another inconclusive finding was that the difference in socioeconomic levels between reported cases citing and not citing abandonment was small. Dog ownership is positively correlated to household income [30,42,52]. However, it has been suggested that people with lower household income are less likely to relinquish their dogs, for financial reasons [14]. Therefore, it has been argued that other variables, including problematic behaviours [15,53,54], nature of the dog [15,22,53], human factors [53,55], and the human–dog bond [14,56] may also play important roles in determining the benefits of dog ownership. This finding again supports the previous assumption that other factors also critically influence some complaint types.

Similarly, we hypothesized that complaints about ‘blood sports’, such as ‘Knowingly allowing an animal to kill / injure another’ and ’Dog fighting or other prohibited offence’ would be associated with lower socioeconomic backgrounds. However, the IRSAD score did not significantly differ between reported cases cited and not cited with this code. Previous research exploring financial aspects of dog-fighting in the UK has pointed out that this kind of ‘blood sporting’ was more popular among working-class men, as a way of life and an alternative expression of masculinity [32]. Nevertheless, a small proportion of middle-class people might also be involved as business owners or as a hobby [32], and thus increase the average socioeconomic scores. In addition, only a small number of cases were reported involving those alleged complaints, with even fewer being confirmed [28]; thereby, it might not be enough to test for statistically significant differences with validity.

Although most complaints were related to socioeconomic disadvantaged people, one complaint type was reported more commonly among more socioeconomically advantaged people. Those with relatively higher socioeconomic levels were more likely to be reported leaving their dogs unattended in a hot car. This finding partially supports the previous study that people with higher socioeconomic level, mainly living in urban areas, are more likely to own cars and take their dogs for outdoor activities [41], and thus have a greater chance of leaving their dogs alone in a car. In high socioeconomic regions and in high density urban regions it would be more likely that owners are reported when they left their dogs in a vehicle in a busy public area.

Although this study reports unconfirmed dog welfare complaints, the results reflect the major welfare concerns in dog populations in higher or lower socioeconomic background. Considering the relevance of different complaint reasons and different socioeconomic levels, intervention strategies for the prevention of animal neglect or cruelty should be directed differently in high and low socioeconomic regions. Studies of confirmed welfare issues are required. Interventions are recommended to be taken in lower socioeconomic areas to explore whether the high number of reports is driven by actual offenses or by higher public awareness. These results can also be used to increase public awareness and promote public education. For instance, councils of relatively higher socioeconomic regions are recommended to place more emphasis on enforcing that people do not leave dogs in a hot vehicle unattended. Councils of relatively lower socioeconomic regions can highlight information for owners on the basic needs of dogs (e.g., the amount of water and food consumption). Another important implication of this study is that it provides information regarding the correlation of socioeconomic backgrounds and the preference for dog types, which could help develop more tailored educational programs that target different populations.

### 4.4. Limitations and Need for Future Research

Several limitations were identified in this study. First, the dataset consisted of cases reported but not confirmed, so results only reflect a likelihood of correlations between socioeconomics and different types of welfare concerns in dogs. Besides, the socioeconomic data was acquired by linking to the postcodes where an alleged welfare concern occurred (used as a proxy for the owners’ postcode), which appeared to be a generalization and might be an ecological fallacy. Therefore, the results should be cautiously interpreted. Future study could try to obtain a more direct measure of socioeconomic status, for example, the household income of each individual. Second, total numbers of residents in each post code are not accurately known. Therefore, we cannot calculate the exact prevalence of each welfare issue in different areas. If the prevalence in certain regions is particularly high, then sampling bias may occur. Third, breed recognition was based on comments made by complainants or trained inspectors, which may not be accurate. Finally, the data was obtained from populations within Queensland, and thus wider geographical generalization should be made cautiously.

## 5. Conclusions

This dataset was analyzed based on reported but not confirmed cases of canine welfare concerns, so the results reflect the tendency rather than fact. Results correlate the socioeconomic level with different dog breeds. The relationships between socioeconomic levels and different complaint types are also identified. Reported dogs of unrecognizable breeds came from postcodes with lower socioeconomic status to those reporting dogs of recognizable breeds. Among RB dogs, reports concerning utility breeds, terriers, and working dogs were more common than dogs reported in socioeconomically disadvantaged areas, but it is not clear to what extent these breed groups are more prevalent in these areas. People living in lower socioeconomic regions were more likely to be reported to be involved in canine welfare concerns, especially neglect-related complaints, and abusing dogs. In contrast, people living in higher socioeconomic areas were more alleged to leave their dogs unattended in a hot vehicle. This study provides detailed information which may help in the development of tailored strategies for different populations to combat welfare concerns in dogs. However, the differences of socioeconomic level were relatively small so the results should be interpreted cautiously. Finally, more risk factors and their roles in different complaint types should also be identified in order to give a better picture of canine welfare concerns.

## Figures and Tables

**Table 1:** Descriptive analysis of Index of Relative Socio-Economic Advantage and Disadvantage (IRSAD) scores of the postcode of dogs in our study and those determined for the population of Queensland.

	Study Group	Queensland	*p* Value (One-Sample Sign Test)
Median	975	1014	<0.001
Q1	935	967	
Q3	1021	1039	

Q1: the first quartile; Q3: the third quartile.

**Table 2:** Index of Relative Socio-Economic Advantage and Disadvantage (IRSAD) scores of the postcode of owners of dogs reported to be recognised breeds (RB) and unrecognised breeds (UB).

	UB (n = 35, 080)	RB (n = 56, 663)	*p* Value (Mann-Whitney Test)
Median	970	981	<0.001
Q1	934	935	
Q3	1014	1024	

Q1: the first quartile; Q3: the third quartile.

**Table 3:** Total numbers and Index of Relative Socio-Economic Advantage and Disadvantage (IRSAD) scores of the postcode of reported dogs in each breed group (*p* < 0.001).

Breed Group	N	Mean ± (SD)	Grouping *				
Gundogs	4394	994 ± (63.0)	A				
Toys	5203	986 ± (64.3)		B			
Hounds	3152	984 ± (63.7)		B	C		
Non Sporting	5056	980 ± (64.6)			C	D	
Working Dogs	14,049	977 ± (63.0)				D	E
Terrier	15,979	977 ± (62.7)					E
Utility	8830	976 ± (62.2)					E

* Means not sharing a letter are significantly different (*p* < 0.05) by Games-Howell pairwise comparisons. SD: standard deviation.

**Table 4:** Median Index of Relative Socio-Economic Advantage and Disadvantage (IRSAD) scores of the postcode of reported dogs for whom each complaint code was used, and the odds ratio and the 95% confidence interval (CI) of the IRSAD score in the logistic regression model of complaint codes. Outcomes of these models were different complaint codes and the fixed factor was the IRSAD score of the owners’ postcode.

Complaint Code	IRSAD Score of the Owner’s Postcode When This Code Cited	IRSAD Score of the Owner’s Postcode When This Code Not Cited	IRSAD Odds Ratio and (CI)	*p* Value
Abandonment	976	975	- ^a^	- ^a^
Dog fighting *	969	975	0.9991 (0.9979, 1.0003)	0.143
Cruel act ^+^	972	975	0.9994 (0.9991, 0.9997)	<0.001
Emergency relief	960	975	- ^b^	- ^b^
Hot animal in car	999	975	1.0067 (1.0063, 1.0071)	<0.001
Insufficient food and/or water	971	979	0.9981 (0.9978, 0.9983)	<0.001
No exercise/confined/tethered	970	979	0.9979 (0.9977, 0.9982)	<0.001
No shelter	973	977	0.9990 (0.9987, 0.9994)	<0.001
No treatment	972	977	0.9986 (0.9983, 0.9988)	<0.001
Overcrowding	968	977	0.9982 (0.9970, 0.9993)	0.002
Poor dog condition	969	979	0.9974 (0.9972, 0.9976)	<0.001
Poor living condition	975	977	0.9996 (0.9993, 0.9998)	0.002

^a^ Not selected by the binary logistic regression model (alpha value to enter > 0.15). ^b^ The IRSAD did not meet the assumption of being linear to the logit so was not included in the binary logistic regression model. * Dog fighting is a combined code including Causing captive animal to be injured/killed by dog, Dog fighting or other prohibited offence, Keeping or using an animal for blooding/coursing a dog, Knowingly allowing an animal to kill/injure another. ^+^ Cruel act is a combined code including Baiting/poisoning, cruelty, Prohibition order breached, Tail docking or other surgical procedure.

## Appendix A

**Table A1:** Description of each complaint code, alleging a welfare issue.

Complaint Code	Description
Abandonment	An animal was abandoned/left by the owner either at their abode or somewhere else such as in the bush.
Baiting/Poisoning	An animal was poisoned or planned to be poisoned.
Causing captive animal to be injured/killed by dog	A person let a captive animal be injured/killed by a dog.
Cruelty	A person was reported to have abused an animal.
Dog fighting or other prohibited offence	A person was reported as allowing dogs to fight or conducting other specifically prohibited acts.
Emergency relief	Emergency relief is required for an animal left unattended because its owner experienced an emergency (e.g., flood or being hit by a car).
Hot animal in car	An animal was left unattended in a car during hot weather.
Insufficient food and/or water	An animal has insufficient food and/or water.
Keeping or using animal for blooding/coursing a dog	A person used a live bait for blooding/coursing a dog.
Knowingly allowing an animal to kill/injure another	A person allows one animal to kill/injuring another one, and does nothing to stop them.
No exercise/confined/tethered	An animal is confined or tethered and not given a suitable amount of exercise.
No shelter	An animal is not provided with suitable shelter provisions.
No treatment	An animal did not receive appropriate medical treatment when needed.
Overcrowding	The number of animals was too high for the living space provided.
Poor dog condition	The general condition of an animal is poor. (e.g., messy/matted coat, pussy eyes, etc.)
Poor living condition	The living environment of the animal is poor.
Prohibition order breached	An owner violated a prohibition order ^a^.
Tail docking or other surgical procedure	Tail docking or other surgical procedure (e.g., declaw removal, etc.) was conducted on an animal.
Unknown	Unknown

^a^ Prohibition order-A prohibition order is given by the court when a person convicted of an animal welfare offense must not possess any or specific animal for a prescribed period of time [8].

## Appendix B

**Table A2:** Breed list.

Breed Reported	Associated Listed Breed	Breed Group
Affenpinscher	Affenpinscher	Toys
Afghan hound	Afghan Hound	Hounds
Airedale terrier	Airedale Terrier	Terrier
Akita	Akita	Utility
Alaskan husky	Siberian Husky	Utility
Alaskan malamute	Alaskan Malamute	Utility
American bulldog	American Bulldog	Non sporting
American foxhound	Foxhound	Hounds
American pit bull terrier	Pit Bull Terrier	Terrier
American Staffordshire terrier	Staffordshire Terrier	Terrier
American water spaniel	American Water Spaniel	Gundogs
Anatolian shepherd dog	Anatolian Shepherd Dog	Utility
Australian bandog	Cross Breed	UB
Australian bulldog	Australian Bulldog	Non sporting
Australian bulldog cross	Australian Bulldog	Non sporting
Australian cattle dog	Australian Cattle Dog	Working dogs
Australian koolie	Coolie/Koolie	UB
Australian sheepdog	Australian Sheepdog	Working dogs
Australian shepherd	Australian Shepherd	Working dogs
Australian silky terrier	Australian Silky Terrier	Toys
Australian stumpy tail cattle dog	Australian Stumpy Tail Cattle Dog	Working dogs
Australian terrier	Australian Terrier	Terrier
Bandogge mastiff	Cross Breed	UB
Basenji	Basenji	Hounds
Basset fauve de bretagne	Basset Fauve De Bretagne	Hounds
Basset hound	Basset Hound	Hounds
Beagle	Beagle	Hounds
Bearded collie	Bearded Collie	Working dogs
Bedlington terrier	Bedlington Terrier	Terrier
Belgian shepherd	Belgian Shepherd	Working dogs
Belgian shepherd-Groenendael	Belgian Shepherd	Working dogs
Belgian shepherd-Laekenois	Belgian Shepherd	Working dogs
Belgian shepherd-Malinois	Belgian Shepherd	Working dogs
Belgian shepherd-Tervueren	Belgian Shepherd	Working dogs
Bernese mountain dog	Bernese Mountain Dog	Utility
Bichon fries	Bichon Frise	Toys
Bloodhound	Bloodhound	Hounds
Bluetick coohound	Bluetick Coohound	Hounds
Border collie	Border Collie	Working dogs
Border collie × Labrador	Border Collie	Working dogs
Border collie, miniature	Border Collie	Working dogs
Border terrier	Border Terrier	Terrier
Borzoi	Borzoi	Hounds
Boston terrier	Boston Terrier	Non sporting
Bouvier des flandres	Bouvier Des Flandres	Working dogs
Boxer	Boxer	Utility
Boxer cross	Boxer	Utility
Boxer × bullmastif	Boxer	Utility
Boxer × American Staffordshire terrier	Boxer	Utility
Bracco Italiano	Bracco Italiano	Gundogs
Briard	Briard	Working dogs
British bulldog	British Bulldog	Non sporting
Brittany	Brittany	Gundogs
Bull Arab	Bull Arab	UB
Bull Arab × greyhound	Bull Arab	UB
Bull terrier	Bull terrier	Terrier
Bull terrier cross	Bull terrier	Terrier
Bull Terrier, Miniature	Bull terrier	Terrier
Bulldog	British bulldog	Non sporting
Bulldog cross	British bulldog	Non sporting
Bullmastiff	Bullmastiff	Utility
Bullmastiff cross	Bullmastiff	Utility
Bullmastiff × wolfhound × Great dane	Bullmastiff	Utility
Cane corso (Italian mastiff)	Cane corso	Utility
Canaan dog	Canaan dog	Non sporting
Cairn terrier	Cairn terrier	Terrier
Cattle dog	Australian cattle dog	Working dogs
Cattle dog cross	Australian cattle dog	Working dogs
Cavalier King Charles spaniel	Cavalier King Charles spaniel	Toys
Central Asian shepherd dog	Central Asian shepherd dog	Utility
Cesky terrier	Cesky terrier	Terrier
Chesapeake bay retriever	Chesapeake bay retriever	Gundogs
Chihuahua	Chihuahua	Toys
Chihuahua cross	Chihuahua	Toys
Chihuahua × Jack Russell	Chihuahua	Toys
Long hair chihuahua	Chihuahua	Toys
Chinese crested dog	Chinese crested dog	Toys
Chinese crested dog—powder puff	Chinese crested dog	Toys
Chow chow	Chow chow	Non sporting
Clumber spaniel	Clumber spaniel	Gundogs
Cocker spaniel	Cocker spaniel	Gundogs
Cocker spaniel, American	Cocker spaniel	Gundogs
Cocker spaniel, English	Cocker spaniel	Gundogs
Collie	Collie	Working dogs
Collie rough	Collie	Working dogs
Collie smooth	Collie	Working dogs
Corgi	Corgi	Working dogs
Corgi, Cardigan Welsh	Corgi	Working dogs
Corgi, Pembroke Welsh	Corgi	Working dogs
Corgi × fox Terrier	Corgi	Working dogs
Coton de tulear	Coton de tulear	Toys
Cross breed	Cross breed	UB
Curly coated retriever	Curly coated retriever	Gundogs
Dachshund	Dachshund	Hounds
Dachshund, long-haired	Dachshund	Hounds
Dachshund, miniature	Dachshund	Hounds
Dalmatian	Dalmatian	Non sporting
Dalmatian cross	Dalmatian	Non sporting
Dandie dinmont terrier	Dandie dinmont terrier	Terrier
Deerhound	Deerhound	Hounds
Dingo	Cross breed	UB
Dingo cross	Cross breed	UB
Dobermann	Dobermann	Utility
Dogue de bordeaux	Dogue de bordeaux	Utility
Dunker	Dunker	Hounds
Dutch shepherd	Dutch shepherd	Working dogs
English foxhound	Foxhound	Hounds
English pointer	English pointer	Gundogs
English mastiff	English mastiff	Utility
English setter	English setter	Gundogs
English springer spaniel	Springer spaniel	Gundogs
English toy terrier	English toy terrier	Toys
Field spaniel	Field spaniel	Gundogs
Finnish lapphund	Finnish lapphund	Working dogs
Flat coated retriever	Flat coated retriever	Gundogs
Formosan mountain dog (Taiwan Dog)	Formosan mountain dog	Utility
Fox Terrier	Fox terrier	Terrier
Fox terrier, smooth	Fox terrier	Terrier
Foxhound	Foxhound	Hounds
French bulldog	French bulldog	Non sporting
German coolie	Coolie/koolie	UB
German hunting terrier	German hunting terrier	Terrier
German pinscher	German pinscher	Utility
German shepherd	German shepherd	Working dogs
German shepherd cross	German shepherd	Working dogs
German shorthaired pointer	German shorthaired/wirehaired pointer	Gundogs
German spitz	Spitz	Non sporting
German wirehaired pointer	German shorthaired/wirehaired pointer	Gundogs
Glen of Imaal terrier	Glen of Imaal terrier	Terrier
Golden retriever	Golden retriever	Gundogs
Gordon setter	Gordon setter	Gundogs
Great dane	Great dane	Non sporting
Great dane × bull Arab	Great dane	Non sporting
Great dane × bullmastiff	Great dane	Non sporting
Great pyrenees	Great pyrenees	Working dogs
Greater Swiss mountain dog	Greater Swiss mountain dog	Working dogs
Greyhound	Greyhound	Hounds
Griffon bruxellois	Griffon bruxellois	Toys
Harrier	Harrier	Hounds
Havanese	Havanese	Toys
Hungarian vizsla	Hungarian vizsla	Gundogs
Husky	Siberian husky	Utility
Husky cross	Siberian husky	Utility
Ibizan hound	Ibizan hound	Hounds
Irish red & white setter	Irish setter	Gundogs
Irish setter	Irish setter	Gundogs
Irish terrier	Irish terrier	Terrier
Irish water spaniel	Irish water spaniel	Gundogs
Irish wolfhound	Irish wolfhound	Hounds
Italian greyhound	Italian greyhound	Toys
Italian spinone	Italian spinone	Gundogs
Jack Russell terrier	Jack Russell terrier	Terrier
Japanese chin	Japanese chin	Toys
Japanese spitz	Spitz	Non sporting
Kangal shepherd dog	Kangal shepherd dog	Utility
Keeshond	Keeshond	Non sporting
Kelpie	Kelpie	Working dogs
Kelpie cross	Kelpie	Working dogs
Kelpie × staffordshire terrier	Kelpie	Working dogs
Kelpie × border collie	Kelpie	Working dogs
Kelpie × cattle dog	Kelpie	Working dogs
Kelpie × labrador	Kelpie	Working dogs
Kelpie × dingo	Kelpie	Working dogs
Kerry blue terrier	Kerry blue terrier	Terrier
King Charles spaniel	King Charles spaniel	Toys
Kuvasz	Kuvasz	Working dogs
Labrador retriever	Labrador retriever	Gundogs
Labrador retriever cross	Labrador retriever	Gundogs
Labradoodle	Labrador retriever	Gundogs
Lagotto Romagnolo	Lagotto Romagnolo	Gundogs
Lakeland terrier	Lakeland terrier	Terrier
Large Munsterlander	Large Munsterlander	Gundogs
Leonberger	Leonberger	Utility
Large terrier cross	Terrier	Terrier
Lancashire heeler	Lancashire heeler	Working dogs
Lhasa apso	Lhasa apso	Non sporting
Louisiana Catahoula leopard dog	Louisiana Catahoula leopard dog	Working dogs
Löwchen	Löwchen	Toys
Lurcher	Cross breed	UB
Maltese	Maltese	Toys
Maltese cross	Maltese	Toys
Manchester terrier	Manchester terrier	Terrier
Maremma sheepdog	Maremma sheepdog	Working dogs
Mastiff	Mastiff	Utility
Mastiff cross	Mastiff	Utility
Mastiff × bull Arab	Mastiff	Utility
Medium terrier	Terrier	Terrier
Medium terrier cross	Terrier	Terrier
Miniature fox terrier	Fox Terrier	Terrier
Miniature pinscher	Miniature pinscher	Toys
Neapolitan mastiff	Neapolitan mastiff	Utility
New Zealand huntaway	New Zealand huntaway	Working dogs
Newfoundland	Newfoundland	Utility
Norfolk terrier	Norfolk terrier	Terrier
North Queensland bullhound	Cross breed	UB
Norwegian elkhound	Norwegian elkhound	Hounds
Norwich terrier	Norwich terrier	Terrier
Nova Scotia duck tolling retriever	Nova Scotia duck tolling retriever	Gundogs
Old English sheepdog	Old English sheepdog	Working dogs
Papillon	Papillon	Toys
Parson Russell terrier	Parson Russell terrier	Terrier
Pekingese	Pekingese	Toys
Peruvian hairless dog	Peruvian hairless dog	Hounds
Petit basset griffon vendeen	Petit basset griffon vendeen	Hounds
Pharaoh hound	Pharaoh hound	Hounds
Pit bull terrier	Pit bull terrier	Terrier
Pig dog	Cross breed	Terrier
Pointer	Pointer	Gundogs
Polish lowland sheepdog	Polish lowland sheepdog	Working dogs
Pomeranian	Pomeranian	Toys
Poodle	Poodle	Non sporting
Poodle toy	Poodle	Non sporting
Poodle miniature	Poodle	Non sporting
Poodle standard	Poodle	Non sporting
Poodle × Shih Tzu	Poodle	Non sporting
Portugese podengo	Portugese podengo	Hounds
Portuguese water dog	Portuguese water dog	Utility
Pug	Pug	Toys
Puli	Puli	Working dogs
Prague ratter	Cross breed	UB
Pyrenean mastiff	Pyrenean mastiff	Utility
Pyrenean mountain dog	Pyrenean mountain dog	Utility
Rhodesian ridgeback	Rhodesian ridgeback	Hounds
Rottweiler	Rottweiler	Utility
Rottweiler × mastiff	Rottweiler	Utility
Russian black terrier	Russian black terrier	Utility
Saint bernard	Saint bernard	Utility
Saluki	Saluki	Hounds
Samoyed	Samoyed	Utility
Sarplaninac	Sarplaninac	Utility
Schipperke	Schipperke	Non sporting
Schnauzer	Schnauzer	Utility
Schnauzer, miniature	Schnauzer	Utility
Schnauzer, standard	Schnauzer	Utility
Schnauzer, giant	Schnauzer	Utility
Scottish terrier	Scottish terrier	Terrier
Sealyham terrier	Sealyham terrier	Terrier
Shar pei	Shar pei	Non sporting
Shar Pei cross	Shar pei	Non sporting
Shetland sheepdog	Shetland sheepdog	Working dogs
Shiba Inu	Shiba Inu	Utility
Shih tzu	Shih tzu	Non sporting
Shih tzu × maltese	Shih tzu	Non sporting
Siberian husky	Siberian husky	Utility
Skye terrier	Skye terrier	Terrier
Sloughi	Sloughi	Hounds
Small terrier cross	Terrier	Terrier
Smithfield cattle dog	Cross breed	UB
Soft coated wheaten terrier	Soft coated wheaten terrier	Terrier
Spaniel	Spaniel	Gundogs
Spanish water dog	Spanish water dog	Gundogs
Spitz	Spitz	Non sporting
Spoodle	Cocker spaniel	Gundogs
Staffordshire bull terrier	American Staffordshire bull terrier	Terrier
Staffordshire bull terrier × labrador	American Staffordshire bull terrier	Terrier
Staghound	Staghound	UB
Swedish vallhund	Swedish vallhund	Working dogs
Tenterfield terrier	Tenterfield terrier	Terrier
Terrier	Terrier	Terrier
Thai ridgeback	Thai ridgeback	Hounds
Tibetan mastiff	Tibetan mastiff	Utility
Tibetan spaniel	Tibetan spaniel	Toys
Tibetan terrier	Tibetan terrier	Non sporting
Timber shepherd	Cross breed	UB
Weimaraner	Weimaraner	Gundogs
Welsh springer spaniel	Springer spaniel	Gundogs
Welsh terrier	Welsh terrier	Terrier
West highland white terrier	West highland white terrier	Terrier
Whippet	Whippet	Hounds
White Swiss shepherd dog	White Swiss shepherd dog	Working dogs
Wirehaired fox terrier	Fox terrier	Terrier
Xoloitzcuintle	Xoloitzcuintle	Non sporting
Yorkshire terrier	Yorkshire terrier	Toys
